# Expression pattern and function of Notch2 in different subtypes of first trimester cytotrophoblast

**DOI:** 10.1016/j.placenta.2015.01.009

**Published:** 2015-04

**Authors:** K. Plessl, S. Haider, C. Fiala, J. Pollheimer, M. Knöfler

**Affiliations:** a Department of Obstetrics and Fetal-Maternal Medicine, Reproductive Biology Unit, Medical University of Vienna, Vienna, Austria; b Gynmed Clinic, Vienna, Austria

**Keywords:** Human placenta, Extravillous trophoblast, Notch2, Migration

## Abstract

**Introduction:**

Notch signalling has been shown to control cytotrophoblast (CTB) proliferation, differentiation and motility suggesting that the conserved signalling pathway could be critical for human placental development. Since individual Notch receptors have not been elucidated, we herein investigated expression pattern and function of Notch2 in different first trimester trophoblast subpopulations.

**Methods:**

Localisation of Notch2 was analysed in first trimester placental and decidual tissues using immunofluorescence. Notch2 transcript and protein levels were studied by qRT-PCR and Western blotting in proliferative EGF receptor (EGFR)^+^ and differentiated HLA-G^+^ CTBs, respectively, isolated from early placentae by MACS. CTB migration through fibronectin-coated transwells as well as proliferation (EdU labelling) in floating villous explant cultures and primary CTBs were investigated in the presence of Notch2 siRNAs or specific antibodies blocking Notch2 cleavage.

**Results:**

In tissue sections Notch2 expression was higher in HLA-G^+^ distal cell column trophoblasts (dCCTs) compared to proximal CCTs. Accordingly, expression of Notch2 mRNA and protein were elevated in isolated HLA-G^+^ CTBs compared to EGFR^+^ CTBs. Notch2 was also detectable in interstitial CTBs as well as in intramural CTBs associated with maternal decidual vessels. Antibody-mediated inhibition of Notch2 signalling did not affect proliferation, but increased migration of SGHPL-5 cells and primary CTBs. Similarly, Notch2 siRNA treatment promoted trophoblast motility.

**Discussion:**

Notch2 is present in differentiated cells of the extravillous trophoblast lineage, such as dCCTs, interstitial and intramural CTBs, suggesting diverse roles of the particular receptor. Notch2 signalling, activated by cell–cell contact of neighbouring dCCTs, could attenuate trophoblast migration.

## Highlights

• Notch2 is induced during EVT differentiation.
• Notch2 in interstitial trophoblasts could regulate cross-talk with decidual cells.
• Notch2 expressed by intramural trophoblasts may play a role in vessel remodelling.
• Notch2 impairs trophoblast migration in vitro.

## 1 Introduction

Human placentation involves the generation of diverse trophoblast subtypes which are essential for subsequent embryonic development. Whereas the villous syncytium produces hormones to maintain pregnancy, anchoring villi attaching to the maternal uterus give rise to extravillous trophoblasts (EVTs) invading decidual stroma and vessels. Interstitial cytotrophoblasts (iCTBs) approach the wall of maternal spiral arteries and promote vessel remodelling in conjunction with uterine NK (uNK) cells and endovascular cytotrophoblasts (eCTBs), the latter replacing maternal endothelial cells [1,2]. These processes are required for continuous, low pressure blood flow to the placenta, thereby ensuring adequate nutrition of the fetus and preventing oxidative stress [3]. Abnormal changes in myometrial vessel remodelling have been detected in pregnancy diseases such as preeclampsia and intrauterine growth restriction [4,5]; their causes however remain largely elusive. Failures in trophoblast proliferation and EVT differentiation could be involved since CTBs, isolated from preeclamptic patients, lack critical lineage marker genes and show changes in gene expression pattern when differentiating in vitro [6,7]. However, our knowledge about the differentiation program of the anchoring villus is only scarce. Whereas many different studies investigated soluble factors regulating trophoblast migration [8,9], numerous key questions concerning placental development, such as mechanisms controlling cell column proliferation and differentiation of progenitors into distinct EVT subtypes, have not been unravelled.

The conserved Notch signalling pathway has been shown to control stem cell renewal, cell fate decisions and differentiation [10,11]. Upon cell–cell contact, membrane-anchored ligands, the Serrate-like ligands (Jagged1 and 2) and the Delta-like ligands (DLL1, 3 and 4), bind to the different Notch receptors (Notch1–4) and thereby provoke activation of the pathway. Subsequent downstream signalling involves two proteolytic cleavage steps executed by members of a disintegrin and metalloproteinase (ADAM) family and γ-secretase finally generating the Notch intracellular domain (NICD) which translocates to the nucleus. Upon binding NICD converts the transcription factor recombination signal binding protein for immunoglobulin kappa J region (RBPJκ) into a transcriptional activator. After recruitment of additional co-activators of the Mastermind-like (MAML) family the NICD-RBPJκ complex induces target genes involved in proliferation, cell lineage determination and differentiation [12].

Recent evidence suggests that the Notch signalling pathway could also be critically involved in placental development and function. Mice harbouring homozygous deletions of different Notch signalling components displayed defects in chorioallantoic branching, labyrinth formation/function or chorion-allantoic fusion [13]. Moreover, *Notch2* mutant mice die around E11.5 due to reduced blood delivery to the placenta and lower numbers of blood sinuses within the labyrinth [14]. Conditional deletion of *Notch2* in progenitors of the invasive trophoblast lineage reduced endovascular invasion, diameter of trophoblast-lined vascular canals and placental perfusion [15]. Hence, functional data as well as the expression pattern of Notch2 and its putative ligands in different trophoblast subtypes [16] suggest a vital role of the receptor in murine placentation.

Similar to mice, Notch signalling could also regulate human trophoblast function and/or differentiation. Indeed, Notch receptors and ligands were detected in the diverse CTB populations of first and second trimester placenta and changes in gene expression between normal and preeclamptic placental tissues were noticed [15,17–19]. Chemical inhibition of γ-secretase and siRNA-mediated gene silencing of *RBPJκ* increased proliferation of villous CTBs (vCTBs) and cell column trophoblasts (CCTs) in first trimester villous explant cultures as well as EVT marker expression in primary CTBs suggesting that canonical Notch signalling could control the differentiation program of anchoring villi [18,20]. The role of individual Notch receptors, however, has not been elucidated in human trophoblasts. To gain more insights into specific functions of Notch we herein analysed the expression pattern of Notch2 in different trophoblast subtypes and studied its role in trophoblast proliferation and EVT migration.

## 2 Materials and methods

### 2.1 Tissue collection

First trimester placental (n = 50) and decidual (n = 5) specimens were gained from elective terminations. Tissues were collected with written informed consent and utilization was approved by the Ethics Committee of the Medical University of Vienna.

### 2.2 Cultivation of primary human first trimester CTBs and SGHPL-5 cells

Primary human CTBs were isolated from first trimester placentae according to a modified protocol of Tarrade et al. [21], as previously published [18,20,22]. Purified trophoblasts were seeded onto fibronectin-coated (20 μg/ml; BD Biosciences, Franklin Lakes, NJ) wells at a density of 5 × 10^5^ cells/24-well. To isolate CTB subtypes, magnetic bead sorting (MACS) was used to separate proliferative, EGFR^+^ CTBs from differentiated, HLA-G^+^ CTBs. EGFR-PE antibody (Santa Cruz Biotechnology, Dallas, TX) or HLA-G-PE antibody (Exbio, Praha, Czech Republic) and anti-PE micro-beads (Miltenyi Biotec, Bergisch Gladbach, Germany) were utilised. SGHPL-5 cells were cultivated in DMEM/Ham's F12, supplemented with 10% FCS and 0.05 mg/ml gentamicin as published [23].

### 2.3 Immunofluorescence analysis

First trimester placental/decidual tissues and floating placental explants were fixed in 7.5% formaldehyde and embedded in paraffin (Merck Millipore, Darmstadt, Germany). Serial sections of these samples were deparaffinised and antigens retrieved in PT Module Buffer 1 (100× citrate buffer, pH 6; Thermo Fisher Scientific) by KOS MicrowaveStation (Milestone Srl, Sorisole, Italy). Sections were incubated with primary antibodies against Cytokeratin-7 (OV-TL 12/13, DAKO, 1:100), Cytokeratin wide spectrum (GeneTex, GTX29377, 1:100), DLL1 (ab76655, Abcam, 1:100), DLL4 (PAB10200, Abnova, 1:100), HLA-G (MEM-G/9, Exbio, 1:100), Jagged1 (H-114, Santa Cruz Biotechnology, 1:100), Jagged2 (C23D2, Cell Signaling, 1:100), Notch2 (D76A6, Cell Signaling, 1:100), HAI-1 (H180, Santa Cruz, 1:100) or VE-Cadherin (BV9, Abcam, 1:100) at 4 °C overnight. Normal Rabbit IgG (Cell Signaling, 1:100) and Rabbit mAb IgG (DA1E, Cell Signaling, 1:250) were used as negative controls (not shown). Subsequently, sections were incubated with goat anti-mouse or anti-rabbit IgG conjugated to Alexa Fluor 488 or Alexa Fluor 568 (2 μg/ml; Molecular Probes, Life Technologies) for 1 h at room temperature. All sections were counterstained with DAPI (1 mg/ml; Roche Diagnostics, Mannheim, Germany). Images were acquired on a fluorescence microscope (Olympus BX50, CC12 digital camera, CellˆP software, Olympus, Hamburg, Germany).

### 2.4 Quantitative RT-PCR

RNA was isolated using peqGOLD TriFast™ (PEQLAB, Biotechnologie GmbH, Erlangen, Germany). Amount and purity were measured with NanoDrop spectrophotometer (ND-1000, PEQLAB). cDNA synthesis, qRT-PCR and data analysis were performed as previously described [24], using 7500 Fast Real-Time PCR System (Applied Biosystems). The FAM™ dye-labelled TaqMan^®^ MGB probes (Applied Biosystems) utilised were EGFR (Hs00193306_m1), HLA-G (Hs00365950_g1), ITGA1 (Hs00235006_m1), ITGA5 (Hs01547673_m1) Notch2 (Hs01050702_m1) and HES1 (Hs00172878_m1). The housekeeping gene TATA-binding protein (TBP; 4333769F) was used as endogenous control.

### 2.5 Western blot analysis

Protein lysates were separated by SDS-PAGE, blotted onto methanol-activated PVDF membranes (GE Healthcare, Buckinghamshire, U.K.) and incubated with primary antibodies against EGFR (D38B1, Cell Signaling, 1:1000), GAPDH (14C10, Cell Signaling, 1:1000), HLA-G (MEM-G/9, Exbio, 1:500), Notch2 (D76A6, Cell Signaling, 1:1000) and TCF-4 (C9B9, Cell Signaling, 1:1000) at 4 °C overnight. Finally, the blots were incubated with HRP-linked anti-mouse (1:25,000; GE-Healthcare) or anti-rabbit (1:5.000; Cell Signaling Technology, Danvers, MA) IgG secondary antibodies for 1 h at room temperature and developed with ECL Prime Western blotting detection reagent (GE Healthcare). The proteins were visualised with MultiImage III FC Light Cabinet and Alpha View 3.1.1.0 software (Alpha Innotech, San Leandro, CA).

### 2.6 siRNA-mediated gene silencing and inhibition of Notch2

For siRNA-mediated silencing primary CTBs were transfected with non-targeting control siRNAs (D-001810-01-05) or Notch2 siRNAs (L-012235-00; ON-TARGETplus SMARTpools, Dharmacon-Thermo Fisher Scientific, Waltham, CA) using the 4D-Nucleofector (program EO-100) and Amaxa SG Cell Line 4D Nucleofector X Kit L (Lonza, Basel, Switzerland). SGHPL-5 cells were transfected by using Lipofectamine RNAiMAX transfection reagent (Life Technologies). For specific inhibition of Notch2 signalling, cultivated cells were treated with 0.4 μg/ml Notch2 blocking antibody or 0.4 μg/ml human IgG isotype control (Novus Biologicals, Littleton, CO). The blocking antibody binds and stabilises the negative regulatory region of the receptor, thereby inhibiting conformational changes, ADAM mediated-cleavage and Notch2-ICD (N2ICD)-dependent activation of canonical signalling [25] as also recently published [26].

### 2.7 Migration assay

24 h after siRNA transfection or treatment with Notch2 blocking antibody cells were seeded onto fibronectin-coated transwells (Merck Millipore, 12 μm pore size for trophoblasts, 8 μm for SGHPL-5 cells). After additional 24 h inserts were fixed with 4% paraformaldehyde and nuclei of migrated cells were stained with DAPI and visualised by fluorescence microscopy. Five non-overlapping pictures per membrane were taken at a 40-fold magnification and digitally analysed with ImageJ software as published [20].

### 2.8 Proliferation assay

Proliferation in placental villous explant cultures and isolated CTBs was analysed by EdU-Click 488 (baseclick GmbH, Tutzing, Germany). Primary cells and floating explants, dissected from first trimester placenta, were cultivated overnight in DMEM/Ham's F12 in the presence of 10 μM EdU and 0.4 μg/ml Notch2 blocking antibody or 0.4 μg/ml IgG isotype control and subsequently processed for immunofluorescence analysis as mentioned [18,20].

### 2.9 Luciferase assay

Primary CTBs and SGHPL-5 cells were transfected with 1 μg/ml of a luciferase reporter plasmid containing four RBPJκ binding sites and 0.5 μg/ml pCMV-β-galactosidase (CMV-βGal, normalisation control) as previously mentioned [20]. Transfected cells were cultivated overnight with the Notch2 blocking antibody. Subsequently, cells were lysed in cell culture lysis buffer (Promega, Madison, WI) containing protease inhibitor cocktail (1:100; Invitrogen-Life Technologies). β-galactosidase and luciferase activities were measured as previously published [18,27].

### 2.10 Statistical analysis

Statistical analysis was performed with Student's *t*-test or Welch's test using SPSS version 17 (IBM Corporation, Armonk, NY). A *p*-value <0.05 was considered statistically significant. Gaussian distribution and equality of variances were determined with Kolmogorov–Smirnov test and Levene's test, respectively.

## 3 Results

### 3.1 Notch2 predominantly localizes to differentiated HLA-G^+^ CTBs

**Supplementary Fig. 1:**
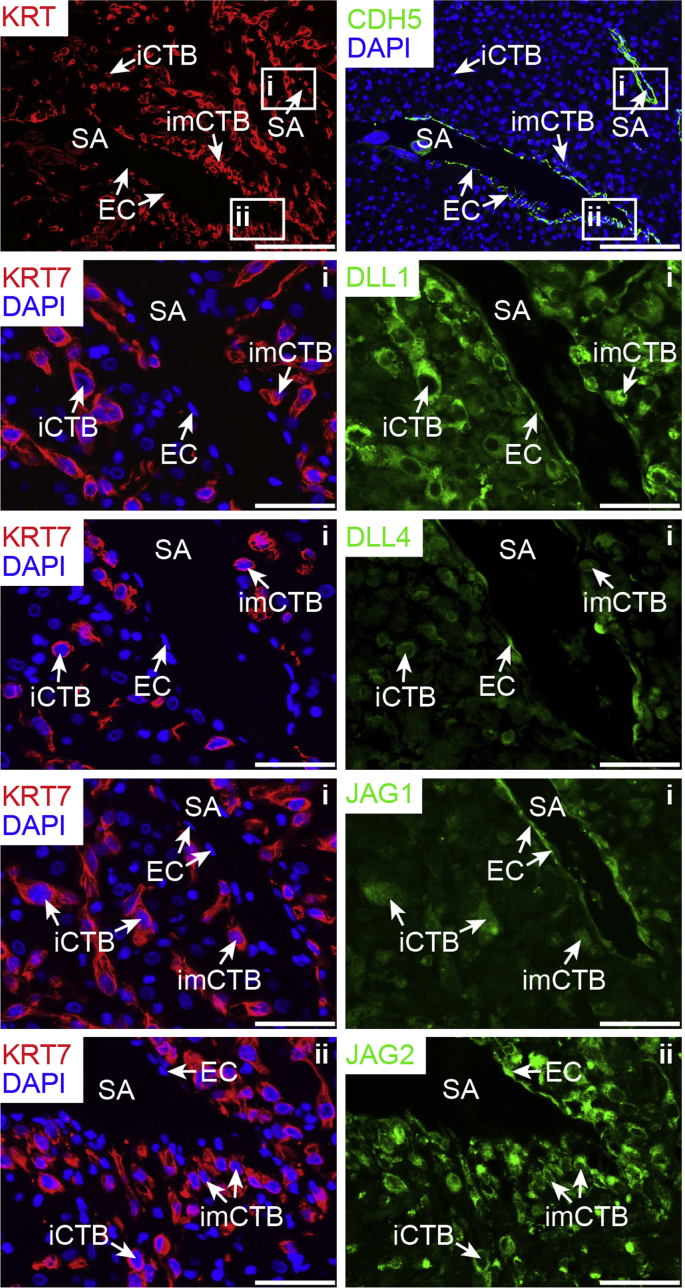
Expression of Notch ligands in first trimester decidua basalis (12th week). Cytokeratin 7 (KRT7) or cytokeratin wide spectrum (KRT) was used as markers for CTB subtypes. Nuclei were stained with DAPI. Representative samples are shown. Pictures labelled with (i) and (ii) represent 400-fold magnifications (scale bar 50 μm) of selected regions of the upper panel (200-fold magnification, scale bar 100 μm). Maternal endothelial cells (EC) of spiral arteries (SA) expressed DLL1, JAG1 and JAG2 and showed a patchy DLL4 expression. The ligands were also expressed by interstitial CTBs (iCTB) and intramural CTBs (imCTB).

**Fig. 1:**
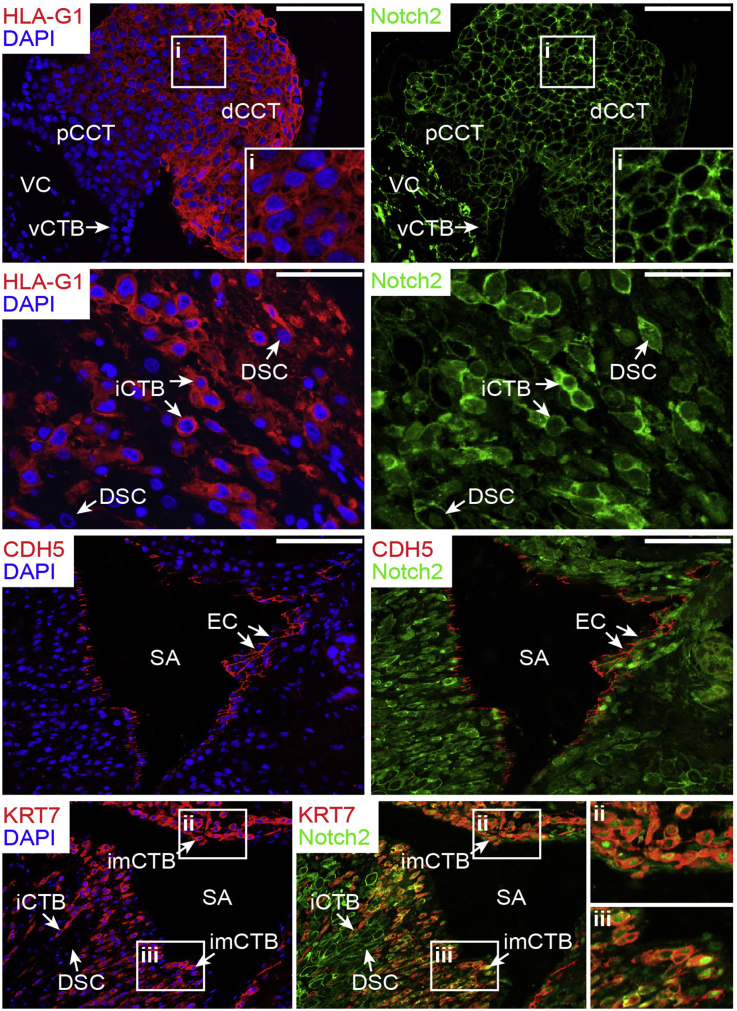
Expression of Notch2 in first trimester placental and decidual tissues. Cytokeratin 7 (KRT7) and HLA-G were used to mark all CTB subtypes and EVTs. Nuclei are stained with DAPI. Representative examples showing localisation of Notch2 in cell column trophoblasts (first panel; dCCT, distal cell column trophoblast; pCCT, proximal cell column trophoblast; 12th week placenta, scale bars represent 100 μm), in interstitial cytotrophoblasts (iCTBs) of the decidua basalis (second panel; 12th week, scale bars represent 50 μm), and in intramural cytotrophoblasts (imCTB), associated with maternal blood vessels (fourth panel; 12th week decidua basalis, digitally zoomed). In all placentae analysed (n = 4, between 6th and 12th week of gestation) expression was weaker in pCCTs and villous cytotrophoblasts (vCTB) compared to non-proliferative, HLA-G^+^ dCCTs. Inserts (i) depict digital magnifications, showing HLA-G and Notch2 in dCCTs. Notch2 was also present in the villous core (VC) as well as in decidual stromal cells (DSC), some of which showed nuclear staining as published [26]. VE-cadherin (CDH5) stained endothelial cells (EC) in spiral arteries (SA) present in maternal decidua (third panel; 12th week decidua basalis, scale bars represent 100 μm). Partial disruption of the maternal EC layer/VE-cadherin staining depicted in (ii) suggested on-going vessel remodelling. imCTBs in (ii) and (iii) showed nuclear Notch2 staining. Selected areas (ii) and (iii) on the right-hand side represent magnified overlays of KRT7^+^/Notch2^+^ imCTBs with nuclear N2ICD.

Immunofluorescence of first trimester placental tissues revealed expression of Notch2 in the villous core, in vCTBs as well as in CCTs (Fig. 1). Notch2 increased in distal areas of the cell column and was also abundantly expressed in migratory HLA-G^+^ interstitial CTBs (iCTBs) of decidua basalis as well as in intramural cytotrophoblasts (imCTBs) surrounding maternal blood vessels (Fig. 1). Several of the imCTBs exhibited nuclear Notch2 staining. Closer inspection of arterial vessels harbouring cytokertatin 7^+^ imCTBs revealed that the adjacent maternal endothelial cell (EC) layer expressed the Notch ligands DLL1, DLL4, JAG1 and JAG2 (Supplementary Fig. 1). DLL1 and JAG2 were also strongly expressed in iCTBs and imCTBs, whereas these CTB subtypes weakly produced DLL4 and JAG1 (Supplementary Fig. 1).

### 3.2 Notch2 expression is associated with EVT differentiation

**Fig. 2:**
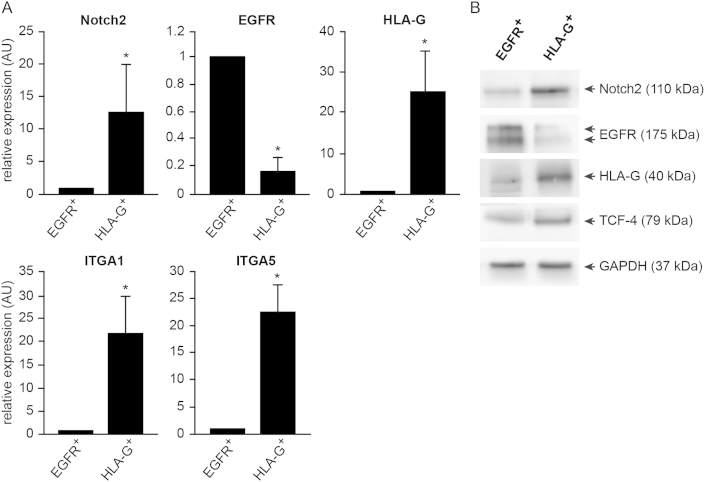
Expression of Notch2 and EVT markers in EGFR^+^ and HLA-G^+^ CTBs isolated from first trimester placental tissue. (A) Transcript levels of *Notch2*, *EGFR*, *HLA-G*, *integrin α1* (*ITGA1*) and *integrin α5* (*ITGA5*) in the two different CTB populations using qRT-PCR. Mean values ± S.D. obtained from five different CTB isolations are shown. For relative quantification (AU, arbitrary units) values of each target gene were arbitrarily set to 1 in EGFR^+^ CTBs. *p < 0.05. Consistent with EVT differentiation *HLA-G*, *ITGA1* and *ITGA5* mRNAs were elevated in HLA-G^+^ CTBs compared to EGFR^+^ CTBs, the latter expressing high levels of *EGFR*. (B) Protein levels of Notch2, EGFR, HLA-G and TCF-4 in the two purified CTB cell types analysed by Western blotting. In agreement with the mRNA expression data HLA-G^+^ CTBs expressed increased levels of Notch2, HLA-G and TCF-4. The latter has been recently established as a marker of EVT [27,29]. GAPDH was used as loading control.

Notch2 was measured by qRT-PCR and Western blotting in EGFR^+^ CTBs and HLA-G^+^ EVTs isolated from first trimester placentae using MACS (Fig. 2). Both *Notch2* mRNA (Fig. 2A) and protein (Fig. 2B) were elevated in the HLA-G^+^ cell pool confirming immunofluorescence data.

### 3.3 Notch2 impairs trophoblast migration

**Supplementary Fig. 2:**
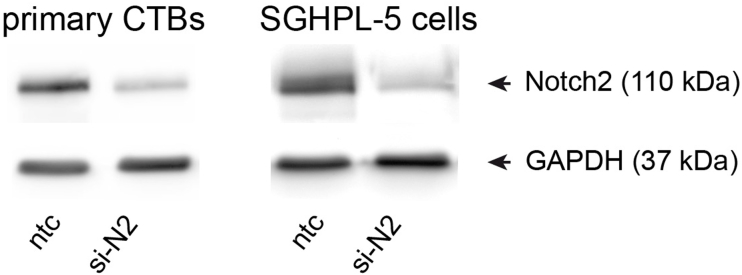
Gene silencing of Notch2 in primary CTBs and SGHPL-5 cells. Notch2 expression was analysed by Western blotting after treatment of cells with specific Notch2 siRNAs (si-N2) or non-targeting controls (ntc) for 48 h. GAPDH was used as loading control.

**Supplementary Fig. 3:**
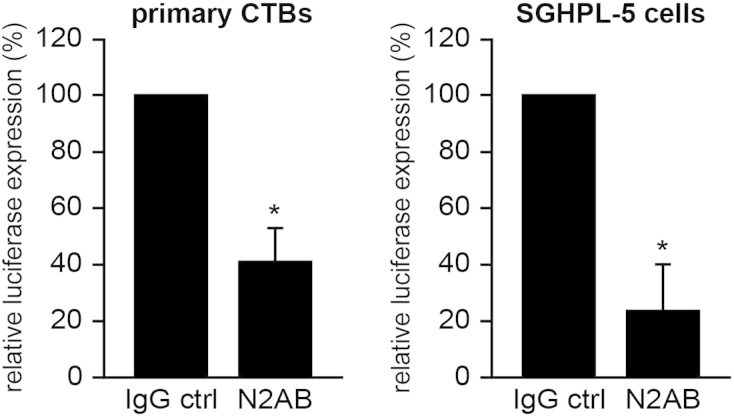
Notch2 antibody-mediated inhibition of canonical Notch reporter activity in primary CTBs and SGHPL-5 cells. Cells were transfected with luciferase reporter plasmids harbouring wild-type RBPJκ cognate sequences and treated with IgG control (IgG ctrl) or Notch2 blocking antibody (N2AB) overnight. Luciferase activity was normalised to constitutive β-Gal expression. Mean values ± S.D. of three different experiments performed in duplicates are shown. Values obtained from IgG control-treated cells were arbitrarily set to 100%. *p < 0.05.

**Fig. 3:**
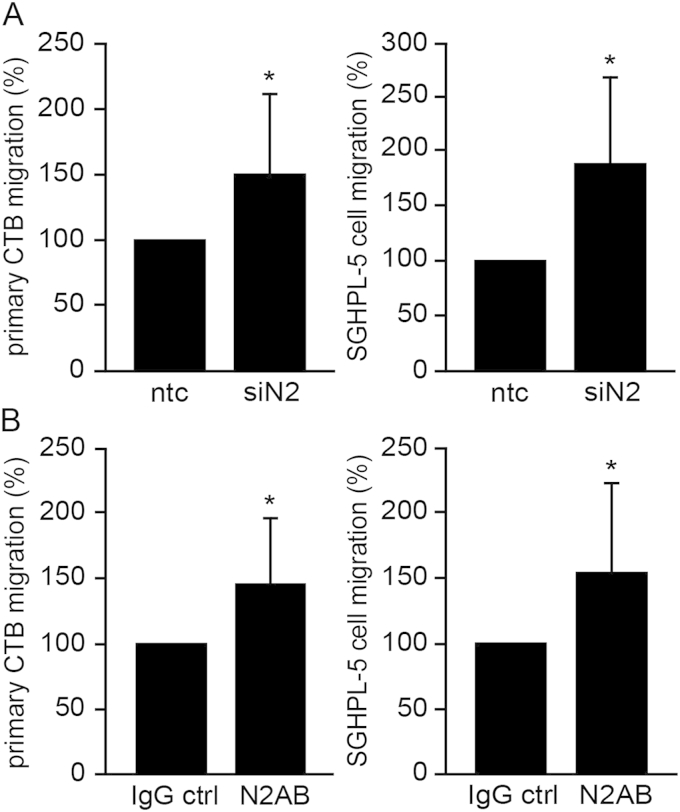
siRNA-mediated Notch2 knockdown or antibody-mediated inhibition of Notch2-dependent signalling increase motility of primary CTBs and SGHPL-5 cells through fibronectin-coated transwells. (A) Migration in the presence of Notch2 siRNA (siN2) or non-targeting control (ntc). Bars represent mean values ± S.D. obtained from each three CTB isolations/SGHPL-5 cell experiments performed in duplicates. ntc was arbitrarily set to 100%. *p < 0.05. (B) Migration upon addition of Notch2 blocking antibody (N2AB) or IgG control (IgG ctrl). Bars represent mean values ± S.D. obtained from three experiments performed in duplicates. IgG control was arbitrarily set to 100%. *p < 0.05.

To assess the role of Notch2 in trophoblast migration (Fig. 3) SGHPL-5 cells and primary first trimester CTBs were treated with Notch2 siRNAs or specific blocking antibodies preventing Notch2 cleavage and therefore N2ICD-dependent transcription. Transfection with Notch2 siRNA decreased Notch2 protein levels in cellular extracts of SGHPL-5 cells and primary CTBs (Supplementary Fig. 2). Efficacy of the Notch2 blocking antibody was confirmed by downregulation of canonical Notch reporter activity in the two cell types (Supplementary Fig. 3). Both siRNA-mediated gene silencing (Fig. 3A) and inhibition of Notch2 cleavage (Fig. 3B) increased migration of primary CTBs and SGHPL-5 cells through fibronectin-coated transwells.

### 3.4 Notch2 inhibition does not affect proliferation

**Supplementary Fig. 4:**
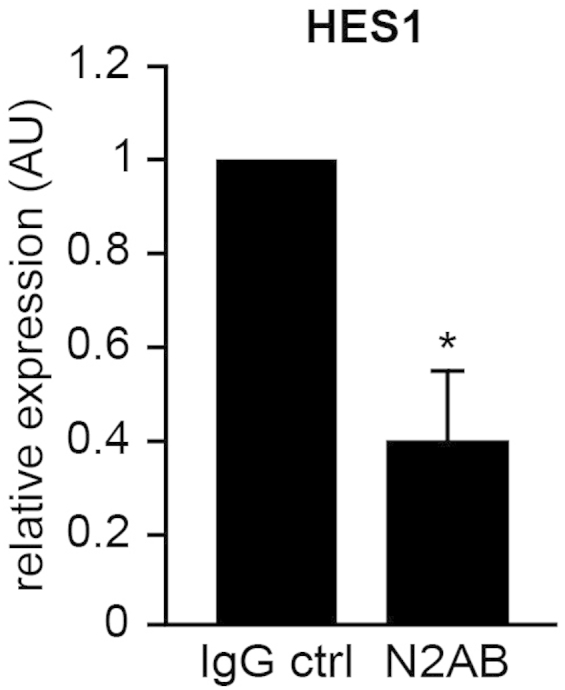
Notch2 blocking in first trimester villous explant cultures inhibits canonical Notch target gene expression. Transcript levels of *HES1*, quantified by qRT-PCR, were significantly decreased in villous explant cultures cultivated with Notch2 blocking antibody (N2AB) for 16 h compared to control conditions (IgG ctrl). Mean values ± S.D. obtained from three different experiments (each 12 placental explants) are shown. For relative quantification (AU, arbitrary units) values of IgG ctrl-treated cultures were arbitrarily set to 1.*p < 0.05.

**Fig. 4:**
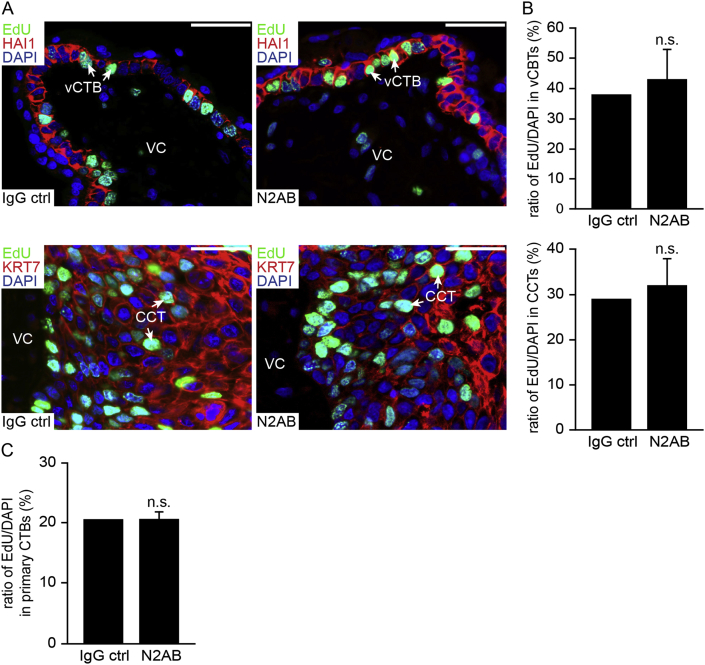
Antibody-mediated inhibition of Notch2-dependent signalling in first trimester floating villous explant cultures and primary CTBs. Proliferation was analysed by EdU incorporation and counting of the EdU/DAPI ratio. (A) Representative pictures (scale bars represent 50 μm) showing immunofluorescent EdU staining in floating explant cultures treated with Notch2 blocking antibody (N2AB) or IgG control (IgG ctrl). Upper and lower panel show EdU labelling in HAI1^+^ vCTBs and KRT7^+^ CCTs. Nuclei are stained with DAPI. EdU^+^ vCTBs and CCTs are indicated by arrows. CCT, cell column trophoblast; VC, villous core; vCTB, villous cytotrophoblasts. (B) Percentage of EdU^+^ villous CTBs and CCTs. Each 14 explants isolated from three different placentae were evaluated in the presence of N2AB or IgG ctrl. Bar graphs represent mean values ± S.D. of 1200–1400 nuclei of vCTBs and 2400–2600 nuclei of CCTs per condition. (C) Percentage of EdU^+^ primary CTBs after treatment with N2AB or IgG ctrl. Bar graphs represent mean values ± S.D. of three experiments performed in duplicates. n.s., not significant.

Advantageous over siRNA treatment, treatment with the blocking antibody allows for immediate inhibition of Notch2-dependent signalling. Hence, proliferation in floating villous explant cultures was assessed in the presence of the inhibitory Notch2 antibody by using immunoflourescent EdU labelling (Fig. 4A). However, evaluation of the percentage of EdU^+^ vCTBs and CCTs cells did not reveal any differences between cultures treated with the blocking antibody or IgG control (Fig. 4B) although efficient downregulation of the canonical Notch target gene *HES1* was observed (Supplementary Fig. 4). Accordingly, inhibition of Notch2 cleavage did neither affect EdU incorporation into isolated primary CTBs (Fig. 4C), nor outgrowth in villous explant cultures seeded on collagen I (data not shown).

## 4 Discussion

Descriptive studies suggest complex roles for Notch2 in the developing mouse placenta. *Notch2* expression varies dynamically throughout mouse gestation and complementary expression of the receptor and its potential interacting ligands have been detected in neighbouring trophoblasts of the ectoplacental cone and labyrinth [16]. Moreover, Notch2 is the predominant receptor in differentiated, invasive trophoblast cell types such as interstitial glycogen trophoblasts and trophoblast giant cells lining spiral arteries and arterial canals [14–16]. Similar to mice, the present study shows that Notch2 expression is mainly associated with differentiated HLA-G^+^ CTB subtypes. Both immunofluorescence in first trimester placental tissues and analyses of primary CTB pools isolated by MACS suggested that Notch2 expression increased during EVT differentiation, the latter being characterised by the induction of marker genes such as *HLA-G*, *ITGA1* and *ITGA5*. Besides elevated expression in HLA-G^+^ dCCTs, Notch2 was also abundant in iCTBs and imCTBs of first trimester decidua basalis. These data are in agreement with previous analyses of second trimester basal plate biopsies showing expression of Notch2 in the different invasive trophoblast cell types [15]. Furthermore, immunofluorescence with the Notch2 antibody, recognizing a membrane-bound, intermediate cleavage product as well as nuclear Notch2, indicated that N2ICD was partly present in imCTB nuclei. The maternal EC layer adjacent to imCTBs expressed JAG1, JAG2, DLL1 and DLL4. Therefore, we speculate that putative binding of maternally-expressed ligands to the Notch2 receptor present on imCTBs could activate canonical, N2ICD-dependent signalling and thereby eventually contribute to remodelling of maternal arterial vessel. In agreement with this assumption, Notch2 was shown to play a crucial role in murine trophoblast invasion and placental perfusion. Conditional deletion of *Notch2* in progenitors of the invasive trophoblast lineage decreased endovascular invasion of spiral arteries and the diameter of maternal canals which deliver blood to the developing placenta [15].

Besides its role in endovascular invasion and remodelling, Notch2 and its interacting ligands could have diverse roles at the placental–decidual interface, likely depending on their respective expression by adjacent interacting cell types. For example, decidual stromal cells, which were shown to control endogenous prolactin and IGFBP-1 expression via Notch2 activation, express DLL1 and DLL4 in a differentiation-dependent manner [26]. The latter ligand impaired in vitro migration of purified CTBs [18]. Moreover, CD45^+^ leukocytes of the decidua produce DLL1, DLL4 and JAG1 [26]. Hence, tissue invasion of iCTBs expressing Notch2 could be controlled by ligands present on interacting decidual stromal cells, macrophages or uterine NK cells. On the other hand, we speculate that ligands expressed by iCTBs could possibly activate Notch2 in stromal cells and thereby control decidual marker gene expression. In this context it is also noteworthy that both DLL1 and DLL4 were shown to activate Notch signalling in decidual NK cells promoting interferon-γ secretion [28].

Moreover, Notch2 could also play a role in the distal cell column where HLA-G^+^ trophoblasts exhibit cell–cell contact before they undergo further differentiation steps along the invasive pathway. Indeed, inhibition of Notch2 cleavage with a specific blocking antibody as well as siRNA-mediated *Notch2* silencing increased migration of SGHPL-5 cells and primary CTBs. These data are in agreement with our recent observation showing that chemical inhibition of γ-secretase, blocking canonical NICD-dependent signalling, also increased EVT motility [18]. Therefore, we speculate that activation of Notch2 by neighbouring HLA-G^+^ CCTs could positively affect cell column integrity and thereby impair trophoblast detachment and migration. Since HLA-G^+^ CCTs express DLL1, DLL4, JAG1 and JAG2 [18], different Notch2-ligand interactions could be involved.

Concurrent with the weak expression of Notch2 in EGFR^+^ CTBs, blocking of N2ICD-dependent signalling did not affect proliferation of CCTs in villous explants cultures and isolated primary cells. In contrast, inhibition of γ-secretase and silencing of *RBPJκ* were shown to increase cell column proliferation and trophoblast outgrowth in these organ cultures [18,20]. The latter treatments also elevated HLA-G, ITGA1, ITGA5 and TCF-4 expression in differentiating CTBs [18,20], whereas these EVT markers were not affected upon antibody-mediated inhibition of Notch2 cleavage (data not shown). Therefore, we assume that the main role of Notch2 is to regulate features of HLA-G^+^ CCTs and different EVT subtypes, whereas other receptors of the proximal cell column, such as Notch1, 3 or 4 [15,18], could control balanced rates of trophoblast proliferation and EVT differentiation. In analogy to that, murine Notch2 was shown to promote endovascular trophoblast invasion but not trophoblast lineage development [14,15]. Depending on the interacting cell type and the expression pattern of ligands Notch2 could regulate column integrity, trophoblast migration, decidual cell function and vessel remodelling.

## Author's contributions

K.P. is a PhD student working on Notch2 signalling in placentation. She performed the majority of the experiments and provided the graphical illustrations. S.H. performed RBPJκ luciferase assays. C.F. provided the placental material and was responsible for clinical management and information of patients. J.P. contributed to study design and writing. M.K. is the principal investigator of the study and wrote the manuscript.

## Conflict of interest

The authors do not report any conflict of interest.

## Disclosure statement

The authors have nothing to declare.

## References

[1] Harris L.K. (2011). IFPA gabor than award lecture: transformation of the spiral arteries in human pregnancy: key events in the remodelling timeline. Placenta.

[2] Pijnenborg R., Vercruysse L., Hanssens M. (2006). The uterine spiral arteries in human pregnancy: facts and controversies. Placenta.

[3] Burton G.J., Jauniaux E., Charnock-Jones D.S. (2010). The influence of the intrauterine environment on human placental development. Int J Dev Biol.

[4] Lyall F., Robson S.C., Bulmer J.N. (2013). Spiral artery remodeling and trophoblast invasion in preeclampsia and fetal growth restriction: relationship to clinical outcome. Hypertension.

[5] Pijnenborg R., Anthony J., Davey D.A., Rees A., Tiltman A., Vercruysse L. (1991). Placental bed spiral arteries in the hypertensive disorders of pregnancy. Br J Obstet Gynaecol.

[6] Lim K.H., Zhou Y., Janatpour M., McMaster M., Bass K., Chun S.H. (1997). Human cytotrophoblast differentiation/invasion is abnormal in pre-eclampsia. Am J Pathol.

[7] Zhou Y., Gormley M.J., Hunkapiller N.M., Kapidzic M., Stolyarov Y., Feng V. (2013). Reversal of gene dysregulation in cultured cytotrophoblasts reveals possible causes of preeclampsia. J Clin Invest.

[8] Knöfler M. (2010). Critical growth factors and signalling pathways controlling human trophoblast invasion. Int J Dev Biol.

[9] Knöfler M., Pollheimer J. (2012). IFPA award in placentology lecture: molecular regulation of human trophoblast invasion. Placenta.

[10] Kopan R., Ilagan M.X. (2009). The canonical Notch signaling pathway: unfolding the activation mechanism. Cell.

[11] Wang Z., Li Y., Banerjee S., Sarkar F.H. (2009). Emerging role of Notch in stem cells and cancer. Cancer Lett.

[12] McElhinny A.S., Li J.L., Wu L. (2008). Mastermind-like transcriptional co-activators: emerging roles in regulating cross talk among multiple signaling pathways. Oncogene.

[13] Gasperowicz M., Otto F. (2008). The notch signalling pathway in the development of the mouse placenta. Placenta.

[14] Hamada Y., Hiroe T., Suzuki Y., Oda M., Tsujimoto Y., Coleman J.R. (2007). Notch2 is required for formation of the placental circulatory system, but not for cell-type specification in the developing mouse placenta. Differentiation.

[15] Hunkapiller N.M., Gasperowicz M., Kapidzic M., Plaks V., Maltepe E., Kitajewski J. (2011). A role for Notch signaling in trophoblast endovascular invasion and in the pathogenesis of pre-eclampsia. Development.

[16] Gasperowicz M., Rai A., Cross J.C. (2013). Spatiotemporal expression of Notch receptors and ligands in developing mouse placenta. Gene Expr Patterns.

[17] Cobellis L., Mastrogiacomo A., Federico E., Schettino M.T., De Falco M., Manente L. (2007). Distribution of Notch protein members in normal and preeclampsia-complicated placentas. Cell Tissue Res.

[18] Haider S., Meinhardt G., Velicky P., Otti G.R., Whitley G., Fiala C. (2014). Notch signaling plays a critical role in motility and differentiation of human first-trimester cytotrophoblasts. Endocrinology.

[19] Herr F., Schreiner I., Baal N., Pfarrer C., Zygmunt M. (2011). Expression patterns of Notch receptors and their ligands Jagged and Delta in human placenta. Placenta.

[20] Velicky P., Haider S., Otti G.R., Fiala C., Pollheimer J., Knöfler M. (2014). Notch-dependent RBPJkappa inhibits proliferation of human cytotrophoblasts and their differentiation into extravillous trophoblasts. Mol Hum Reprod.

[21] Tarrade A., Lai Kuen R., Malassine A., Tricottet V., Blain P., Vidaud M. (2001). Characterization of human villous and extravillous trophoblasts isolated from first trimester placenta. Lab Invest.

[22] Fock V., Mairhofer M., Otti G.R., Hiden U., Spittler A., Zeisler H. (2013). Macrophage-derived IL-33 is a critical factor for placental growth. J Immunol.

[23] Choy M.Y., St Whitley G., Manyonda I.T. (2000). Efficient, rapid and reliable establishment of human trophoblast cell lines using poly-L-ornithine. Early Pregnancy.

[24] Saleh L., Otti G.R., Fiala C., Pollheimer J., Knöfler M. (2011). Evaluation of human first trimester decidual and telomerase-transformed endometrial stromal cells as model systems of in vitro decidualization. Reprod Biol Endocrinol.

[25] Wu Y., Cain-Hom C., Choy L., Hagenbeek T.J., de Leon G.P., Chen Y. (2010). Therapeutic antibody targeting of individual Notch receptors. Nature.

[26] Otti G.R., Saleh L., Velicky P., Fiala C., Pollheimer J., Knöfler M. (2014). Notch2 controls prolactin and insulin-like growth factor binding protein-1 expression in decidualizing human stromal cells of early pregnancy. PLoS One.

[27] Meinhardt G., Haider S., Haslinger P., Proestling K., Fiala C., Pollheimer J. (2014 May). Wnt-dependent T-cell factor-4 controls human extravillous trophoblast motility. Endocrinology.

[28] Manaster I., Gazit R., Goldman-Wohl D., Stern-Ginossar N., Mizrahi S., Yagel S. (2010). Notch activation enhances IFNgamma secretion by human peripheral blood and decidual NK cells. J Reprod Immunol.

[29] Pollheimer J., Loregger T., Sonderegger S., Saleh L., Bauer S., Bilban M. (2006). Activation of the canonical wingless/T-cell factor signaling pathway promotes invasive differentiation of human trophoblast. Am J Pathol.

